# Antibodies to S2 domain of SARS-CoV-2 spike protein in Moderna mRNA vaccinated subjects sustain antibody-dependent NK cell-mediated cell cytotoxicity against Omicron BA.1

**DOI:** 10.3389/fimmu.2023.1266829

**Published:** 2023-11-21

**Authors:** Corey A. Balinsky, Le Jiang, Vihasi Jani, Ying Cheng, Zhiwen Zhang, Tatyana Belinskaya, Qi Qiu, Tran Khanh Long, Megan A. Schilling, Sarah A. Jenkins, Karen S. Corson, Nicholas J. Martin, Andrew G. Letizia, Robert D. Hontz, Peifang Sun

**Affiliations:** ^1^ Henry Jackson Foundation for the Advancement of Military Medicine, Bethesda, MD, United States; ^2^ Leidos, Reston, VA, United States; ^3^ Vysnova Partners, Inc., Landover, MD, United States; ^4^ Virology and Emerging Infectious Department, U.S. Naval Medical Research Unit SOUTH, Lima, Peru; ^5^ Diagnostics and Surveillance Department, Naval Medical Research Command, Silver Spring, MD, United States; ^6^ US Naval Medical Research Unit-INDO PACIFIC, Singapore, Singapore; ^7^ U.S. Naval Medical Research Unit Eurafcent, Sigonella, Italy

**Keywords:** SARS-CoV-2, Moderna mRNA vaccine, ADCC, S2, Omicron BA.1, IgG subclass

## Abstract

Vaccination with the primary two-dose series of SARS-CoV-2 mRNA protects against infection with the ancestral strain, and limits the presentation of severe disease after re-infection by multiple variants of concern (VOC), including Omicron, despite the lack of a strong neutralizing response to these variants. We compared antibody responses in serum samples collected from mRNA-1273 (Moderna) vaccinated subjects to identify mechanisms of immune escape and cross-protection. Using pseudovirus constructs containing domain-specific amino acid changes representative of Omicron BA.1, combined with domain competition and RBD-antibody depletion, we showed that RBD antibodies were primarily responsible for virus neutralization and variant escape. Antibodies to NTD played a less significant role in antibody neutralization but acted along with RBD to enhance neutralization. S2 of Omicron BA.1 had no impact on neutralization escape, suggesting it is a less critical domain for antibody neutralization; however, it was as capable as S1 at eliciting IgG3 responses and NK-cell mediated, antibody-dependent cell cytotoxicity (ADCC). Antibody neutralization and ADCC activities to RBD, NTD, and S1 were all prone to BA.1 escape. In contrast, ADCC activities to S2 resisted BA.1 escape. In conclusion, S2 antibodies showed potent ADCC function and resisted Omicron BA.1 escape, suggesting that S2 contributes to cross-protection against Omicron BA.1. In line with its conserved nature, S2 may hold promise as a vaccine target against future variants of SARS-CoV-2.

## Introduction

The SARS-CoV-2 Spike (S) protein is the primary target for mRNA-based vaccines and therapeutics that effectively prevent severe disease and limit adverse clinical outcomes of COVID-19 (1–3). The S glycoprotein is comprised of two subunits: S1 and S2. The N-terminus S1 (residues 14-685), is further separated into the N-terminus domain (NTD; residues 14-305) and the receptor-binding domain (RBD; residues 319-541). The C-terminus S2 (residues 686-1273) contains a fusion peptide (residues 788-806) and two heptapeptide repeat sequences (HR1 912-984, HR2 1163-1213), enriched with HPPHCPC repeats with hydrophobic residues. The polybasic furin protease cleavage site, which contains a four amino acid insertion, PPAR (aa 681-684), is positioned at the boundary of S1 and S2 (1, 4).

While S1 and RBD delivered by mRNA vaccines can elicit potent neutralizing antibodies that are regarded as key correlates of protection (5–7), and novel vaccines based on the single RBD domain have been developed (8), the role of S2 is less well understood. Several studies have investigated antibody responses to overlapping linear peptides of S2 and revealed broadly neutralizing monoclonal antibodies to conserved B-cell epitopes (9–12). Others have identified conserved CD4 and CD8 T cell epitopes in S2 (13–15). Additionally, S2 vaccines elicited antibodies capable of mediating potent antibody-dependent cell cytotoxicity (ADCC) (16) in mice. These studies suggested that S2 may play a crucial role in the prevention and treatment of disease, but the mechanisms of immune protection have not been well characterized.

Compared to S1, S2 is more conserved among endemic human beta coronaviruses, including OC43 and HKU-1, and alpha coronaviruses, including 229E and NL63 (17). S2 is also highly conserved among all SARS-CoV-2 variants of concern (VOCs), including Alpha (B.1.1.7), Beta (B.1.351), Gamma (P.1), Delta (B.1617.2), and Omicron (B.1.1.529) (18). The VOC with the greatest number of nucleic acid changes when compared to the ancestral strain is Omicron and its related strains. Omicron BA.1 has >30 changes in S1, but only six changes in S2 (19, 20).

Most of the amino acid changes in the RBD of Omicron BA.1 have been mapped to the receptor-binding motif (RBM), the site of the S protein that interacts with ACE-2 (21–24). Nucleic acid changes to RBD led to immune escape, diminished vaccine efficacy, and rendered monoclonal antibody (Ab) therapies ineffective (23–25). Recently, Omicron sub-variants, including BA.2, BA.3, BA.4, BA.5, and BA.1/BA.2, and the latest XBB series, have evolved nucleic acid changes that likely aid in escaping adaptive immunity from vaccinations or previous infections, including earlier infections with variant BA.1 (26–28). Therefore, the identification of non-RBD-targets that are selected based upon S-specific design and can provide a conserved target for pan-coronavirus vaccines are needed for protection against contemporary SARS-CoV-2 threats.

We previously evaluated IgG and neutralizing antibody responses to Omicron BA.1, BA.2, and BA.4/5 SARS-CoV-2 variants in 562 US military members vaccinated with the primary 2-dose series of Moderna mRNA-1273 in a cross-sectional study (29). We found nearly all vaccinated participants had sustained spike (S) IgG and neutralizing antibodies (ND50) to the ancestral strain, but the responses to Omicron BA.1 were reduced by LOGs (29). At 6 months post-vaccination, around 90% of the study participants did not have detectable ND50 to Omicron BA.1 (29). The vaccine efficacy (VE) of mRNA vaccines against symptomatic diseases to Omicron has been shown to decrease to as low as 20.7% by 6 months after the primary two-dose series (30), however, the VE for other clinical endpoints, including the need for supplemental oxygen usage or death, remained above 70% (31, 32). Investigation of protective mechanisms other than antibody neutralization is important for improving vaccine strategies that will remain effective against the ever-growing list of SARS-CoV-2 variants. Antibodies with strong FcR function have been shown to control HIV in humans and SIV in non-human primates (33, 34), and in other diseases such as influenza (35) and Herpes simplex type 2 virus (36). Their role in controlling SARS-CoV-2 requires evaluation.

Utilizing the samples described in the previous study (29), we investigated domain-specific antibody responses to address questions including mechanisms for Ab neutralization and FcR function. We first profiled four IgG subclasses: IgG1, IgG2, IgG3, and IgG4 to RBD, NTD, S1, and S2 domains. We then characterized Ab neutralization and ADCC on different domains by depleting RBD antibodies and by making pseudoviruses bearing domain-specific mutations resembling the BA.1 variant. The results of our study indicate that S2 is a potent antigenic domain for ADCC. Unlike S1, S2 antibodies showed a much lower level of Omicron BA.1 escape.

## Materials and methods

### Serum samples

The Survey of Immune Response to Coronavirus Disease 2019 Infections (SIM-COVID) study is a cross-sectional serological study conducted by the U.S. Naval Medical Research Unit TWO (29). U.S. DoD active-duty members of the Navy and Marines serving in the U.S. Indo-Pacific Command Area of Responsibility (USINDOPACOM AOR) were enrolled. Ethics review was conducted by the Institutional Review Board, Naval Medical Research Center (NMRC IRB) (HRPP#: NAMRU2.2020.0002) in compliance with all applicable federal regulations governing the protection of human subjects. Blood was collected and processed for serum. Antibody to the Spike (S) by ELISA and neutralization antibody titers were determined by a pseudovirus assay as described in our previous publication (29).

The SIM-COVID study enrolled 562 subjects between Feb and Sep of 2021 who had completed two doses of the Moderna mRNA1273 vaccine 0-230 days previously. Upon completion of the cross-sectional study on the SIM-COVID population (29), we observed a significant decrease in antibody responses to Omicron BA.1. The focus of this study was to characterize the polyclonal serum antibodies to address a few basic questions related to immune escape. Therefore, the criterion for sample selection was based on the antibody ELISA and neutralization measured previously to ensure that all samples for this study had positive titers. Samples from 48 subjects meeting the inclusion criteria were used for various assays at random for this study. The median days between the last dose of vaccine to sample collection was 41 (range 11-196). S protein IgG ELISA median endpoint titer was 409600 (range 6400 to 6553600). The neutralizing antibody titers to the vaccine strain assessed by a pseudovirus constructed with S of ancestral strain (mutation D614G) ranged from 442 to 68652, with a median of 3672 (29) (**Table 1**). Due to the depletion of sample volume with each subsequent assay, we were unable to use the same samples for all experiments. However, some samples that had larger volumes were included in as many assays as possible allowing us to do correlation analysis.

**Table 1:** Demographics of the study population.

Sex: number (%)	Male: 36 (75%)
	Female: 12 (25%)
Race: number (%)	Native Hawaiian: 1 (2.1%)
	American Indian: 1 (2.1%)
	African American: 11 (22.9%)
	Asian: 1 (2.1%)
	White: 17 (35.4%)
	Unreported: 17 (35.4%)
Age: Median (range)	27 (21-44)
Days from the 2nd dose of vaccination: Median (Range)	41 (11-196)
ELISA Endpoint: Median (Range)	409600 (6400-1638400)
PD50: Median (Range)	3372 (442-68652)
Period sample collection	03Feb2021 to 10Sep2021
PCR positive: number (%)	0 (0%)

### Quantitative ELISA for IgG subclasses

Antigens used for ELISA were acquired from ACROBiosystems and the ancestral strain sequence refers to GenBank #: QHD43416, sequence published 18-Mar-2020. Omicron BA.1 sequence refers to GISAID clade: GR/484A; Nextstrain Clade: 21K (37). The antigens included RBD (ancestral) (SPD-C52H3), RBD (Omicron BA.1) (SPD-C522e), NTD (ancestral) (S1D-C52H6), NTD (Omicron BA.1) (SPD-C522d), S1 (ancestral) (S1N-C52H3), S1 (Omicron BA.1) (S1N-C52Ha), S2 (ancestral) (S2N-C52H5), and S2 (Omicron) (S2N-C52Hf).

ELISA was performed as previously described (38). Briefly, 1×phosphate buffered saline (1×PBS) was used as Coating Buffer; 1×PBS with 0.1% Tween 20 (1×PBST) was used as Wash Buffer; 5% Difco Skim Milk in 1×PBST was used as Blocking Buffer and Sample Dilution Buffer; IMMULON 4HBX 96-well, flat-bottom microplates (Thermo; Cat: 3855) were used as assay plates. Serum samples were heat-inactivated at 56°C for 1 h and assayed at 1:100 or 1:1,000 dilution. For IgG1, IgG2, IgG3 and IgG4 standard curves, microplates were coated with 1 µg/ml (0.1 ug/well) mouse anti-human IgG-Fc (Southern Biotech Cat #: 9040-01) at 4°C overnight. After washing in 1×PBST, plates were blocked with Blocking Buffer at 37°C for 1 h. Purified Ig subclasses (all from Athens Research & Technology), IgG1 (Cat #:16-16-090707-1M), IgG2 (Cat#:16-16-090707-2M), IgG3 (Cat #: 16-16-090707-3) and IgG4 (Cat #: 16-16-090707-4M), were 2-fold serially diluted with Sample Dilution Buffer as standard curves (ranging from 0.4 ng/ml to 3200 ng/ml). The plates were then incubated at 37°C for 1 h. After washing in 1×PBST, the plates were incubated with 1:1,000 diluted mouse anti-human IgG1-HRP (ThermoFisher Cat # A10648), mouse anti-human IgG2-HRP (Southern Biotech, Cat #:9060-05), mouse anti-human IgG3-HRP (Southern Biotech, Cat#:9210-05), and mouse anti-human IgG4-HRP (Southern Biotech, Cat:9200-05) at 37°C for 1 h. HRP was detected with TMB Microwell Peroxidase substrate (ThermoFisher, Cat:5120-0077), and the reactions were stopped by 1N sulfuric acid (H2SO4) solution. Plates were read at OD450nm on a plate reader (PerkinElmer EnSpire) within 30 min of stopping. We subtracted responses against the non-specific binding (PBS-coated wells) during data finalization to yield antigen-specific responses. IgG1, IgG2, IgG3, and IgG4 concentrations (ng/ml) of each sample were calculated by their sigmoidal standard curves using GraphPad Prism 9.0.2.

### Domain-specific pseudovirus

Pseudovirus (PV) was produced using the SARS-CoV-2 Spike (S) gene and the lentivirus-derived reporter and packaging plasmids previously described by Crawford et al., 2020, and generously made available through BEI resources (NIAID, NIH). Wuhan strain with the D614G mutation (referred to as the ancestral strain), and Omicron BA.1 (B.1.1.529) S gene sequences, were obtained from the GSAID database (www.gsaid.org) and codon optimized for human expression, with 19 amino acids removed from the C-terminus. Chimeric spike proteins for the study of domain-specific neutralization were generated using the ancestral strain and switching specified domains for sequenced derived from Omicron BA.1 [NTD (aa 14-315), RBD (aa 331-528) and S2 (aa 690-1257)]. Genes were synthesized by GenScript (Piscataway, NJ) and cloned into pcDNA3.1+ expression vectors. PV neutralization assays were performed as previously described (Balinsky et al., 2022). In brief, serum was heat inactivated at 56°C for one hour prior and sample serum was diluted 1:60 in DMEM containing 10% FBS, followed by three-fold serial dilutions. Assays were then read using a FACS Canto II equipped with an HTS running BD FACSDiva software (Version 8.0.1). Data was analyzed using GraphPad’s Prism software (Version 8.3.1), using nonlinear regression analysis to calculate ND50.

### Depletion of RBD antibodies

The recombinant SARS-CoV-2 recombinant RBD domain of the Spike protein was produced and purified in Expi293F cells (39). The affinity column immobilized with either RBD or bovine serum albumin (BSA) proteins was prepared using AminoLink Coupling Resin (ThermoFisher Scientific). Briefly, one milliliter of recombinant RBD protein (4.7 mg/ml) or 1 milliliter of BSA (4.7 mg/ml) were dialyzed against a coupling buffer containing 0.1M sodium phosphate and 0.15M sodium chloride (pH 7.2). Two chromatography columns (Bio-Rad Laboratories) were each packed with 0.5 ml of AminoLink Coupling Resin and followed by equilibration with 10 10-column volume (CV) of water and subsequently with 10-15 CV of coupling buffer. One milliliter of the dialyzed RBD or BSA protein was added to the column followed by 10 µl of 5M sodium cyanoborohydride (NaCNBH3) solution. The columns were inverted three times to mix and incubated overnight at 4°C on a rocking shaker. The columns were drained and the flow through was collected for residual protein concentration determination (the coupling efficiencies were >90% for both proteins). Columns were then washed with 5 CV of coupling buffer followed by 5 CV of quenching buffer (1M TRIS-HCl, pH 7.4). A half ml of quenching buffer and 10 µl of NaCNBH3 solution were added to each column, mixed, and incubated at room temperature for 30 min on a rocking shaker. The columns were drained and washed with 10 CV of wash buffer (1M NaCl) followed by equilibration with 10-15 CV of binding buffer (1xPBS, pH7.4). To deplete RBD-specific antibodies, 200 µl of human serum sample was added to the column and allowed to enter the resin completely. Subsequently, 500 µl of binding buffer was added to the column and fractions were collected (this process was repeated 3-4 times). The columns were washed with 10-15 CVs of binding buffer before being used for the next serum sample. Protein concentration was determined by Bradford assay and fractions with higher protein concentration were used for downstream analysis.

### Competition assay

Ten samples with measurable ND50 were randomly selected for competition assay. The sera were diluted to one concentration that gave 60-85% neutralization. Briefly, PV was incubated with media or with serum diluted to a single dilution to assess the percent neutralization at this serum dilution. To compete with the neutralization, BSA (Sigma Aldrich) 1ug/well, NTD (AcroBiosystem S1D-C52H6) 1 ug/well, RBD (AcroBiosystem SPD-C52H3) 0.001 ug/well, and RBD 0.001 ug + NTD 1 ug per well were added. Reduction of antibody neutralization was evaluated by comparing percent neutralization with and without competing antigens.

### Plate bound domain specific ADCC

Plate-bound NK cell assay using 96-well plates was adopted from previous publications (40, 41). Briefly, U-bottom 96-well plates (Corning) were coated with 0.2 µg/well of antigens at 4°C overnight together with PBS as a non-specific control. Antigen-coated plates were washed and blocked with Complete Media for 1 hour at 37°C. Then, serially diluted sera were added to allow antibodies to bind to antigens, and unbound serum was removed by washing after 1 hour. Frozen and thawed PBMCs were placed in Primaria cell culture dishes (Corning) to remove adherent cells as described (42). The non-adherent PBMCs were collected and incubated in 10 ng/ml of IL-2 (R&D Systems) at 10^6^ cells/ml in Complete Media (RPMI 1640 supplemented with 10% FBS (Cytiva), 1% Penicillin/Streptomycin (Quality Biological)) overnight. PBMCs treated with recombinant IL-2 (R&D Systems) were washed and added to the plates at 10^5^ cells/well and incubated for one hour at 37°C in a CO_2_ incubator. Golgi-plug (BD Biosciences) was added at 1:1000 and PBMCs were incubated for an additional hour. The cells were washed and stained with an NK cell cocktail: CD3/CD56/CD16/CD107a (BD Biosciences). Subsequently, the cells were fixed with Cytofix and Cytoperm (BD Biosciences), and stained intracellularly with IFNγ (BD Biosciences, Cat# 564791) and TNFɑ (Biolegend, Cat# 502930) as described previously (43). Cytokine expression was determined as %cytokine+ per total CD3-CD56+ cells. Antigen-specific responses were calculated by subtracting responses in antigen-coated wells from uncoated (PBS) wells.

### Stable cell line expressing ancestral and Omicron S

HEK293 cells (ATCC, Cat# CRL-1573) were maintained in DMEM (ThermoFisher) supplemented with 10% FBS (Cytiva) and 1% Penicillin/Streptomycin mixture (Quality Biological) referred to as complete medium DMEM (CM-DMEM). Transfection was performed with plasmids pcDNA3.1 containing Spike (described above) from the parental and Omicron BA.1 using lipofectamine 3000 (ThermoFisher Scientific). At 48 hours post-transfection, selection media consisting of CM-DMEM supplemented with 400 ug/ml G418 (Invivogen) was used to select transfected cells. The cells were then sorted using the WOLF Cell sorter and allowed to recover before adding selection media. Transfected cells were assessed for spike expression using human IgG1 Anti SARS-CoV-2 NTD Antibody (Native Antigen Company, Cat# AM121) as primary and Anti -Human IgG Fc Specific -PE as secondary (Invitrogen) antibody.

### ADCC against full-length S expressed on HEK293 cells

As previously described for plate-bound domain-specific ADCC (41), PBMCs were plated the day before on Primaria cell culture dishes for 1.5 hours (Corning) after which the non-adherent fraction was collected, counted, and resuspended to 10^6^ cells/ml. Next, 10 ng/ml of IL-2 (R&D Systems) was added and the cells were allowed to recover overnight in a humidified incubator at 37°C and 5% CO_2._ The next day, HEK293 cells expressing full-length spike were harvested by removing the media, washing gently with PBS, and adding 3 ml of 5 mM EDTA. The cells were washed, resuspended in DMEM-CM, and added to round bottom 96-well plates (Corning) at 5X10^4^ cells/well. Serum dilutions were then added to the respective wells and incubated on ice for 1 hour. The cells were washed twice with PBS to remove any unbound serum.

For degranulation assays, PBMCs were washed to remove IL-2 from media, counted, and added to the plate with 5X10^4^ cells/well, and incubated for 1 hour at 37°C. Golgi-plug (BD Biosciences) was then added at a final dilution of 1:1000 and the PBMCs were incubated for one hour. The cells were then washed twice with PBS. Surface expression of CD107a on CD3^-^CD56^+^ NK cells was determined using the same antibody panel described in plate-bound ADCC assays above. Controls of the assay included HEK-S cells not treated with serum and HEK cells not transfected with S gene. The NK cell activity to the ancestral S and Omicron BA.1 S was obtained by subtracting the values from controls. Three pre-pandemic samples were used as baseline controls for setting the cut-off (44).

### Statistics

Data were analyzed using GraphPad Prism 8.0 software. Descriptive analyses were used to describe variables. The Shapiro-Wilk test was used to assess the data’s normality. Student T-tests, Friedman, or Kruskal-Wallis tests were used as appropriate for the analysis of significant differences between study groups. The Spearman’s rank correlation coefficient was used to test the correlation between factors and outcomes. A P-value of 0.05 was applied to all statistical analyses.

## Results

### Subclasses of IgG to RBD, NTD, S1 and S2

**Figure 1:**
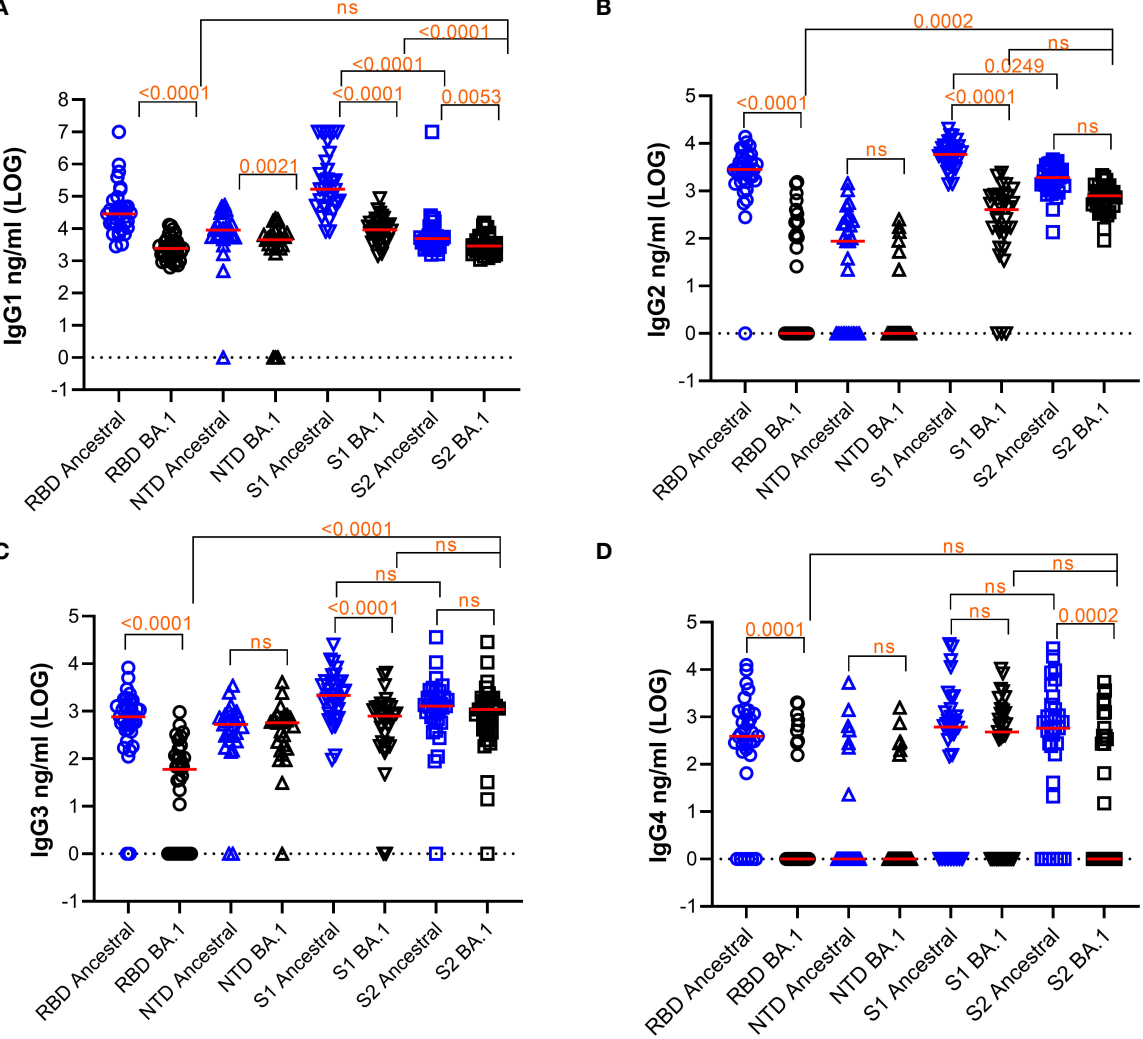
Quantification of subclasses of IgG Abs against various S protein domains of the ancestral strain or Omicron BA.1. Titers of IgG subclasses, IgG1 **(A)**, IgG2 **(B)**, IgG3 **(C)**, and IgG4 **(D)**, and the response median were shown. The dotted line indicates cut-off based on data from pre-pandemic samples. Freidman Dunn’s Multiple Comparison tests were used to compare differences between selected data pairs marked with the bracket and p values. Group median (LOG), range (LOG), and % negative responders (undetected) are summarized in the table below. ns, not significant.

We measured IgG1, IgG2, IgG3, and IgG4 subclasses to RBD, NTD, S1, and S2 of ancestral and Omicron BA.1 antigen using 33 samples (**Figure 1**). The median titers of the responses and the % of samples that fell below detection are shown in **Table 2**. Friedman Dunn’s test was used to compare the differences between the selected data sets.

**Table 2:** LOG pg/ml IgG subclasses (Median, Min, and Max).

Subclass IgG		RBD Ancestral	RBD BA.1	NTD Ancestral	NTD BA.1	S1 Ancestral	S1 BA.1	S2 Ancestral	S2 BA.1
IgG1	Median	4.457	3.381	3.957	3.655	5.222	3.959	3.689	3.458
	Min-Max	3.452-7	2.797-4.115	0-4.723	0-4.317	3.906-7	3.16-4.936	3.196-7	3.041-4.18
	% undetected	0	0	4.0	12	0	0	0	0
IgG2	Median	3.452	0	1.944	0	3.767	2.61	3.281	2.896
	Min-Max	0-4.139	0-3.188	0-3.163	0-2.403	3.137-4.307	0-3.383	2.13-3.66	1.959-3.331
	% undetected	3.0	57.6	37.0	77.8	0	9.1	0	0
IgG3	Median	2.884	1.778	2.723	2.759	3.335	2.899	3.11	3.038
	Min-Max	0-3.915	0-2.982	0-3.539	0-3.61	1.978-4.401	0-3.808	0-4.556	0-4.459
	% undetected	6.1	33.3	8.0	4.0	0	6.1	3.0	3.0
IgG4	Median	2.594	0	0	0	2.787	2.681	2.765	0
	Min-Max	0-4.096	0-3.305	0-3.724	0-3.198	0-4.537	0-4.018	0-4.445	0-3.738
	% undetected	18.2	72.7	74.0	74.0	27.3	36.4	21.2	57.6

Comparing the responses among 4 subclasses, IgG1 was the most abundant subclass for all the antigens tested with the median titer being 1-3 LOGs higher than the rest of the subclasses on average. Reduction of antibody response to BA.1 relative to the ancestral antigens was seen across all subclasses of IgG.

For IgG1, responses to all BA.1 antigens were significantly decreased relative to the ancestral antigens (**Figure 1A** and **Table 2**). Median antibody titers to BA.1 RBD and S1 were reduced by 1.1 and 1.2 LOG (p<0.0001), respectively. The reduction was smaller for BA.1 NTD (0.30 LOG) (p=0.0021) and S2 (0.23 LOG) (p=0.0053). Among the ancestral antigens, the response to S2 was significantly lower than that to S1 by 1.53 LOG (p<0.0001) and RBD by 0.75 LOG (p<0.0001). Among the BA.1 antigens, the response to S2 was significantly lower than S1 by 0.4 LOG (p<0.0001). All subjects had detectable antibodies except a small percentage of subjects were negative to NTD.

For IgG2 (**Figure 1B**), median RBD antibody titer to BA.1 was reduced to 0 (p<0.0001), and 57.6% of samples did not show detectable titer. Median NTD antibody titer to BA.1 reduced to 0 and 37% and 77.8% samples were below detection for ancestral and BA.1 antigens, respectively. Relative to the ancestral antigens, titers to BA.1 S1 reduced by 1.15 LOG (p<0.0001), whereas titers to BA.1 S2 reduced by 0.4 LOG which was not significant (p>0.05). Among the ancestral antigens, the S2 response was significantly less than S1 (0.49 LOG) (p=0.0249). Among the BA.1 antigen, S2 titers were significantly higher than RBD (2.896 LOG) (p=0.002) and comparable to S1(p>0.05).

For IgG3 (**Figure 1C**), antibody titers to RBD of BA.1 were reduced by 1.11 LOG relative to the ancestral RBD. (p<0.0001), and 33.3% of samples showed no detectable antibody. Titers to S1 of BA.1 were reduced by 0.44 LOG (p,0.0001) relative to the ancestral S1. Titers to S2 of BA.1 were comparable to those of ancestral (p>0.05). Among the ancestral antigens, S2 did not show lower titers than RBD or S1, which was different compared to that of IgG1 and IgG2. Among the BA.1 antigens, S2 titer was greater than RBD (0.23 LOG) (p=0.0002) and comparable to S1 (0.2 LOG) (p>0.05).

For IgG4 (**Figure 1D**), the percentages of samples to ancestral and BA.1 RBD (18.2% and 72%), NTD (74% and 74%), S1 (27.3% and 36.4%), and S2 (21.2% and 57.6%) fell below the detection limit.

Overall, the pattern of subclass responses between protein subdomains appeared to be different between RBD, S1, and S2 antigens: S2 did not elicit as much of an IgG1 response when compared to RBD and S1 but did elicit a similar IgG3 response. RBD responses, followed by S1 responses, were prone to significant escape by Omicron BA.1 whereas S2 responses exhibited less escape. Overall, S2 is a competent and dominant antigen recognized by IgG subclasses in subjects vaccinated with mRNA1273 particularly when assessing BA.1.

### The effect of domain-specific amino acid changes on Ab neutralization

**Figure 2:**
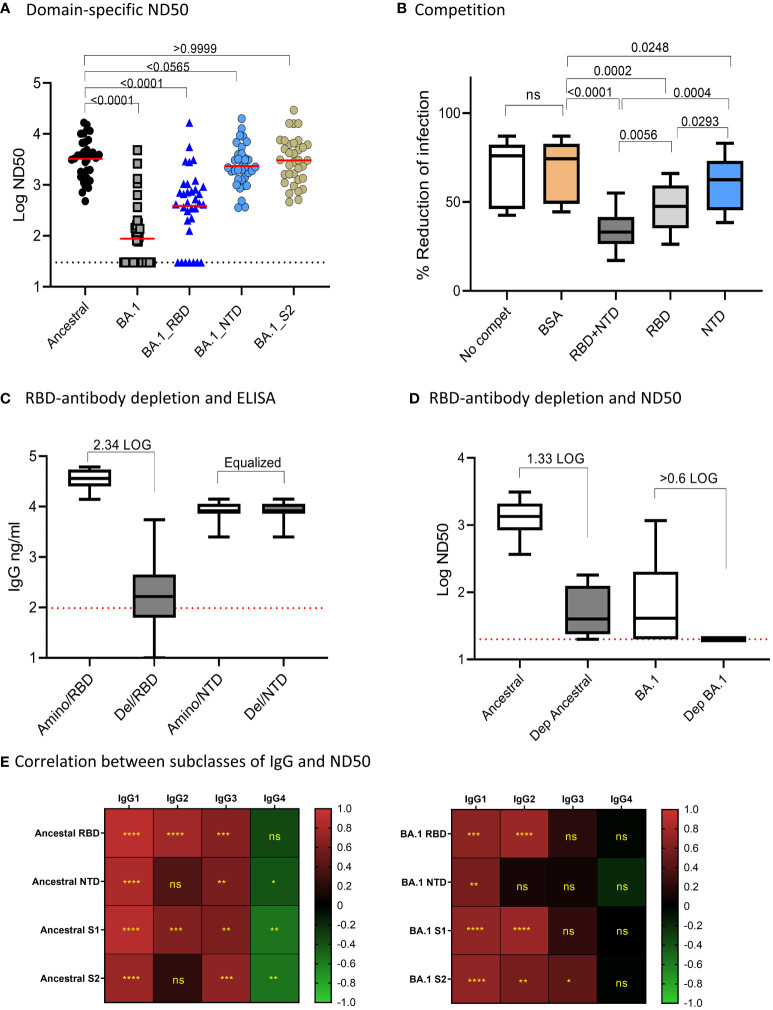
Domain-specific neutralization, competition, and RBD-antibody depletion. **(A)** Domain-specific pseudovirus neutralization: median of the data sets were shown. Freidman Dunn’s Multiple Comparison tests were used to compare differences between data sets marked with brackets and p values are shown. **(B)** Competition assay showing “no competition,” and neut with serum samples pre-incubated with indicated proteins. Data are shown in boxplots and passed Shapiro-Wilk normalization tests followed by RM one-way ANOVA Turkey’s multiple comparison tests. Significant differences between comparing groups and p values are shown. **(C)** Efficiency for depletion of RBD antibodies is shown. Amino represents depletion control using BSA. RBD del represents RBD-antibody depletion. ELISA was performed on RBD and NTD and boxplots are shown for 10 samples. Samples were normalized by NTD antibodies. The times of RBD antibody reduction were calculated by dividing ELISA means of undepleted to depleted samples. The dotted line was the baseline level of RBD response from pre-pandemic samples. **(D)** Neutralization activities to ancestral strain and Omicron in control and RBD-antibody-depleted serum samples. Times of reduction between ND50 in depleted samples and undepleted samples were calculated by dividing the mean of ND50 between the two data sets. The dotted line shows the limit of detection at 60. E-F: Correlation (Spearman correlation) between IgG subclasses and ND50 to the ancestral strain **(B)** and Omicron BA.1 **(C)** are shown as the heatmaps. The asterisks show significant correlations (<0.05). ns, not significant.

We next assessed the impact of amino acid changes on different domains of BA.1 S protein for antibody neutralization (**Figure 2A**). We used 34 randomly selected samples and tested Ab neutralization on the Ancestral and BA.1 pseudoviruses, and on pseudoviruses bearing BA.1 amino acid changes specific for RBD, NTD, and S2 domains on a backbone of the ancestral S protein. The median of ND50s for the ancestral, BA.1, BA.1_RBD, BA.1_NTD, and BA.1_S2 were 3603, 30, 439.4, 2221, and 3057, respectively. BA.1 showed a significant escape of Ab neutralization as compared to that of the ancestral strain (p<0.0001 by Friedman Dunn’s multiple comparison test). Changes to the RBD domain resulted in the greatest escape as compared to changes to other domains. For NTD, the amino acid changes resulted in a 38.4% reduction to the NTD-BA.1 pseudovirus neutralization relative to the ancestral, although not found to be significant (p=0.0565) by Friedman Dunn’s multiple comparison test). Amino acid changes to S2 did not affect Ab neutralization (p>0.9999 by Friedman Dunn’s multiple comparison tests, p=0.8395 by Wilcoxon matched-pairs signed rank test). The domain-specific neutralization data suggested that amino acid changes on RBD were primarily responsible for the escape of BA.1 neutralization, NTD contributed to BA.1 escape to a lesser degree than RBD, and S2 did not contribute to BA.1 escape.

We further performed antigen-competition assays to examine the role of RBD and NTD domains on antibody neutralization. We used ten samples to test the competition of RBD and NTD domains against antibody neutralization to the ancestral strain (**Figure 2B**). We anticipated that if a domain on the pseudovirus was involved in Ab neutralization then adding soluble protein of this domain would compete and reduce neutralization. The median neutralization of the 10 samples at a single serum dilution without competition was 76% and adding BSA minimally affected the neutralization (74%). In the presence of RBD+NTD, RBD, and NTD, the median neutralization became 33%, 48%, and 63%, respectively. In this single serum dilution assay, NTD (median neutralization=62.5%) and RBD (median neutralization=47.5%) alone significantly reduced neutralization as compared to BSA control (median neutralization=76.1%). The combination of RBD and NTD (median neutralization=33%) demonstrated the greatest competition against Ab neutralization. The data suggested that RBD is primarily responsible for Ab neutralization, while NTD played a much lesser role.

We next assessed antibody neutralization on samples depleted of RBD-binding antibodies (**Figures 2C, D**). We chose ten samples randomly and performed RBD antibody depletion. Since the depletion process altered the volume of the samples, we adjusted the sample volume between the depleted and undepleted samples to yield the same NTD antibody concentration. After normalization, the mean NTD antibody concentration between the undepleted and depleted samples was both 8,270 ng/ml. The median RBD antibodies in the undepleted and depleted samples were 36,219 ng/ml and 166 ng/ml, respectively, showing a 2.34 LOG depletion of RBD antibodies (**Figure 2C**). The mean of antibody neutralization (ND50) to the ancestral strain was reduced from 1347 to 41 in undepleted and depleted samples, showing a 1.62 LOG reduction in neutralization after depletion or RBD binding antibodies. Antibody neutralization to BA.1 (median 78.49 before depletion) decreased to below detection after depletion of RBD-antibodies (**Figure 2D**).

Pearson correlation analysis showed that Ab neutralization (ND50) to both the ancestral and Omicron BA.1 correlated with overall Ab binding (**Figure 2E**). The highest positive correlation was with IgG1 and IgG2, followed by IgG3. IgG4 showed negative or no correlation with ND50.

### NK cell activation

**Figure 3:**
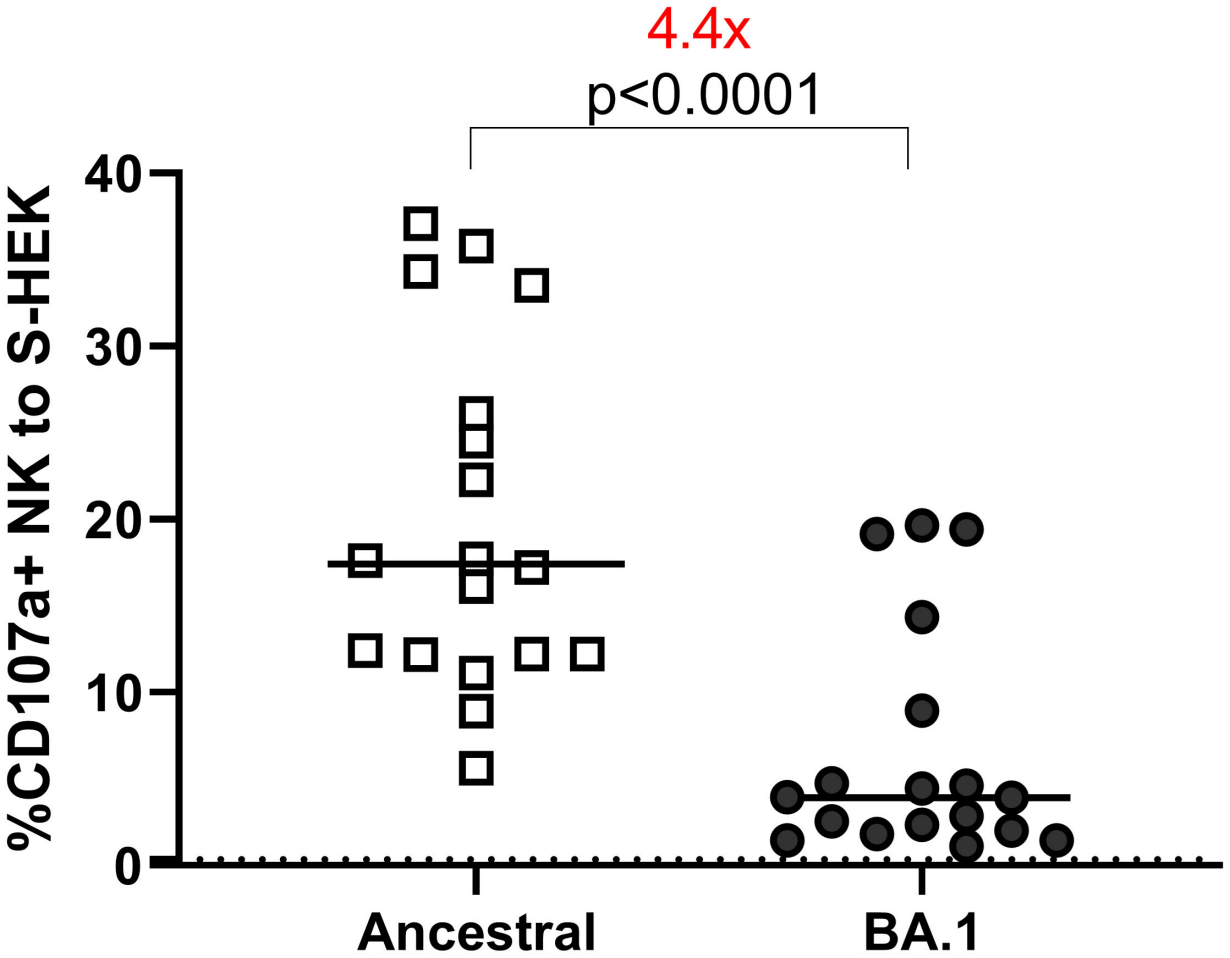
ADCC to S-HEK293 cells. ADCC performed on HEK-293 cells expressing S proteins of ancestral and Omicron BA.1 strains. The Y-axis shows the percentage of the CD107a^+^ NK cells out of the total NK cell (CD3^-^CD56^+^) population. The dotted line shows the cut-off based on the pre-pandemic controls.

We assessed ADCC using cell lines expressing the S protein from the ancestral or BA.1 variants on the cell surface (**Figure 3**). All samples were tested at 1:100 dilution. Eighteen samples were used to determine overall ADCC activities in S-expressing cell lines. The median of the CD107a+ (degranulation) NK responses to the ancestral and BA.1 S were 17.43 and 3.93, respectively. The reduction of NK cell response to Omicron was 4.4 times relative to the ancestral S (p<0.0001 by Wilcoxon test) (**Figure 3**).

### Domain-specific antibodies for activation of NK cells

**Figure 4:**
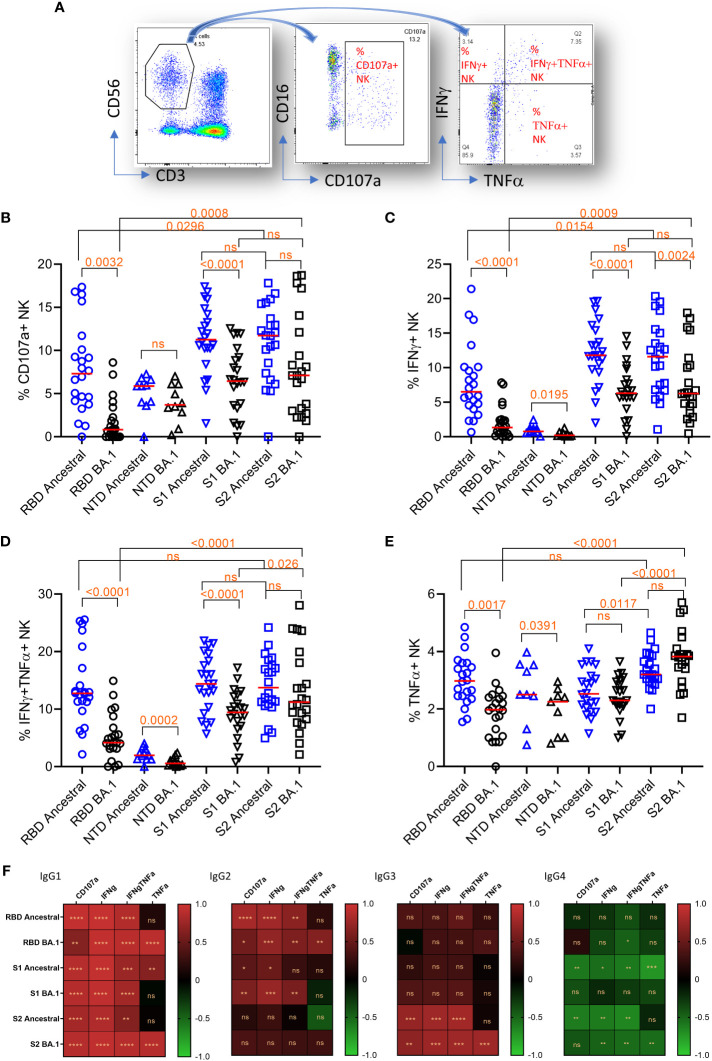
Plate-bound ADCC. **(A)** The gating method for gating on total NK cells (CD3^-^CD56^+^)(left panel), subgating of total CD3-CD56+ cells on CD16 and CD107a (middle panel) to yield the % of CD107a+NK cells, and subdivision of the total CD3-CD56+ cells on IFNγ and TNFα (right panel) to yield the % of single and double cytokine-expressing NK cells. **(B–E)** NK cell responses and marker expression to different antigens (x-axis). Freidman Dunn’s Multiple Comparison tests were used to compare differences between data pairs marked with the bracket. Significant differences are marked with p values. **(F)** Correlation (Spearman correlation) between titers of IgG subclasses and NK markers to the ancestral strain and Omicron BA.1 are shown as the heatmap. The asterisks show significant correlations (<0.05). ns, not significant.

We further measured NK cell degranulation marker CD107a and cytokine expression in NK cells using the plate-bound ADCC assay. For cytokines IFNγ and TNFα, NK cells were segregated into IFNγ or TNFα single positive, or IFNγ/TNFα double positive groups (**Figure 4A**).

The median NK cell degranulation (% CD107a+ NK/total NK cells) response to different antigens were 7.3 (ancestral RBD), 0.8 (BA.1 RBD), 5.9 (ancestral NTD), 3.7 (BA.1 NTD), 11.3 (ancestral S1), 6.5 (BA.1 S1), 11.7 (ancestral S2), and 7.1 (BA.1 S2) (**Figure 4B**). By Friedman Dunn’s multiple comparison (p values shown in the figure), the reduction of CD107a+ response to RBD and S1 of BA.1 relative to the corresponding ancestral antigens was significant. The responses to S2 were not statistically different between the ancestral and BA.1. The response to S2 of BA.1 was significantly higher than that to RBD of BA.1.

The median IFNγ-single positive NK cells (% IFNγ+ NK/total NK cells) were 6.5 (ancestral RBD), 1.3 (BA.1 RBD), 0.8 (ancestral NTD), 0.2 (BA.1 NTD), 11.8 (ancestral S1), 6.3 (BA.1 S1), 11.6 (ancestral S2), and 6.3 (BA.1 S2) (**Figure 4C**). The reduction of the responses to Omicron BA.1 relative to Ancestral for RBD and S1 was significant. No significant difference was observed between responses to ancestral and BA.1 S2. Again, the response to BA.1 S2 was significantly higher than that to BA.1 S1 and RBD.

The median IFNγ/TNFα double positive NK cells (% IFNγ+TNFα+ NK/total NK cells) were 12.7 (ancestral RBD), 4.2 (BA.1 RBD), 2.0 (ancestral NTD), 0.6 (BA.1 NTD), 14.4 (ancestral S1), 9.5 (BA.1 S1), 13.7 (ancestral S2), and 11.2 (BA.1 S2) (**Figure 4D**). The reduction of double IFNγ/TNFα responses to BA.1 relative to ancestral for RBD, NTD, and S1 was significant. No significant difference was seen in S2 between ancestral and BA.1. Again, the response to S2 of BA.1 was significantly higher than that to S1 and RBD of BA.1.

The median TNFα single positive NK cells (% TNFα+ NK/total NK cells) were 3.0 (ancestral RBD), 2.0 (BA.1 RBD), 2.5 (ancestral NTD), 2.3 (BA.1 NTD), 2.5 (ancestral S1), 2.3 (BA.1 S1), 3.2 (ancestral S2), and 3.8 (BA.1 S2) (**Figure 4E**). The reduction of the single-TNFα responses to RBD of BA.1 relative to RBD of ancestral was significant. No significant reduction was seen on S1 and S2 of BA.1. Again, the response to S2 of BA.1 is significantly higher than that to S1 and RBD of BA.1.

We further analyzed the correlation between domain-specific ADCC and IgG subclasses (**Figure 4F**). IgG1 showed the best correlation for all NK cell response markers. IgG2 showed a positive correlation with NK cell response markers for RBD and S1. IgG3 showed a positive correlation with NK cell activities only for S2. IgG4 showed a significant negative correlation with NK cell activity.

Overall, ADCC activity to RBD and S1 were prone to escape by Omicron BA.1. In contrast, ADCC to S2 did not show significant escape for three of the four markers. When comparing different antigens, higher Omicron BA.1 S2 ADCC activities corresponded with sustained IgG subclass responses to S2.

## Discussion

When Omicron variants became the predominant VOCs, after Dec 2021, the population that had completed a mRNA vaccination series against the ancestral strain was significantly less likely to develop severe disease (45, 46). While multiple studies showed subjects with two doses of mRNA vaccines had little measurable neutralizing antibodies to the Omicron variants (23, 47, 48), the mechanism behind antibody-mediated protection provided against severe disease remained unclear. In this study, we characterized Ab subclasses, neutralization, and FcR functions to S1, including RBD and NTD subdomains, and S2 domains of the SARS-CoV-2 ancestral strain and the Omicron BA.1 variant.

Our results showed that the average quantities of IgG subclasses in subjects vaccinated with mRNA-1273 were in the order of IgG1>IgG2-3>IgG4 for all the antigens tested. Therefore, the antibody response among the young adult subjects was consistent with what has been previously reported (49, 50) in other vaccinated subjects.

We compared antibody binding among RBD, NTD, S1, and, S2 antigens. The pattern of ELISA subclasses suggested RBD, S1, and S2 differentially elicit Ig subclasses: S1 and RBD induce more IgG1 and IgG2 whereas S2 more IgG3. Our data on S1 and S2 is consistent with what has been reported after natural infection, in that S1 and S2 elicit an imbalanced subclass response (51). IgG3 is one of the earliest IgG subclasses to appear upon viral infection possibly due to the location of the IgG3 gene within the heavy chain locus (52). As the class switch continues, IgG1 progressively dominates the subclass response (52). IgG3 is regarded as the most functional subclass for Fc binding (52). Switching IgG1 monoclonal antibodies to IgG3 has been shown to enhance Fc-mediated opsonization (53). A monoclonal Ab targeting RBD, when engineered with the IgG3 Fc exhibited up to 50-fold greater neutralization potency as compared to Fc of the other subclasses (54). Studies in mice showed that the S2 domain alone elicited greater ADCC Abs than that of the pre-fusion or full-length S (16, 55). When comparing vaccine antigen targets among mice between S, S1, and S2, the mice immunized with S2 showed the highest antibody response (55, 56). Our data showed that S2 elicited comparable IgG3 and ADCC activities (NK cell degranulation and cytokine production) as compared to S1. Although there is no direct evidence that ADCC protects from viral infection, ADCC has been associated with recovery from severe COVID-19 (16), better HIV vaccine efficacy (57), and slower HIV progression (58, 59). ADCC antibodies can be found in SARS-CoV-2 infected (60) and mRNA vaccinated subjects (61). Our data suggests that S2 is an important component for immune protection but with different working mechanisms from S1 through non-neutralizing ADCC antibodies and cell-mediated immunity. T cells play a role in orchestrating Ig class switching. T cells targeting individual peptide epitopes of S protein differ in expression of memory phenotype, chemokine receptors, and TFH markers (46). Whole blood cells from human subjects vaccinated with CoronaVac, mRNA/CoronaVac prime-boost, or mRNA vaccine secreted greater amounts of IL2 and IFNγ to overlapping peptides covering S2 than S1 (47). While research is needed to demonstrate that cell-mediated immunity participates in protective mechanisms, additional research is also required to characterize T cell responses to S1 and S2 and their role in driving domain-specific IgG subclass selection.

Our ELISA and ADCC data showed that Omicron BA.1 Abs to S2 were comparable to S1 but more abundant than Abs to RBD. We showed that IgG subclass responses to S2 resisted Omicron BA.1 escape more than RBD and S1. We also showed that amino acid changes on S2 did not impact Ab neutralization. These observations are likely due to several differences between S2 and S1. First, there are fewer amino acid changes (6 changes) on S2 as compared to RBD (15 changes) and S1 (32 changes). Second, S2 shares higher sequence conservation and cross-reactivity between SARS-CoV-2 and other endemic beta-coronaviruses than S1 (62–64). We previously showed that infection of SARS-CoV-2 can boost cross-reactive responses to S2 of OC43 and HKU1 but not S1 (65), suggesting the existence of broadly cross-reactive memory B cells to S2. The conserved target of S2 among beta-coronaviruses may make the S2 response more rapid and durable than S1. A S2 vaccine for MERS-CoV was shown to protect mice from lethal infection (66), and monoclonal Abs obtained from these mice passively cross-protected mice from SARS-CoV-2 lethal infection. Finally, while 10 out of the 15 amino acid changes on RBD are mapped to the receptor binding motif (RBM), none of the six amino acid changes on BA.1, A710V/V1176F/T716I/T1027/D1118H were mapped to the conserved epitope regions covering the fusion peptide and the hinge regions of S2.

Immunodominant regions on S2 were mapped to residues 764–829 that overlap the fusion peptide and the S2 cleavage site (R815) and conserved neutralizing epitopes were found in this region (10). A linear epitope on S2 residues 818-843 covering part of the fusion peptide was detected in SARS-CoV-2 convalescent subjects, and depletion of Abs to this epitope resulted in a significant reduction of overall serum neutralizing titer (9). Additional antigenic regions on S2 residues 1148–1159 link HR1 and HR2 and IgG targeting this epitope were detected in 90% of COVID-19 patients (10). Since we did not perform S2-antibody depletion, our results do not exclude the possibility that some anti-S2 antibodies could be neutralizing. Combined with the conservative nature of S2 and its ability to elicit higher levels of IgG3, future vaccine design which induces a more balanced S1 and S2 immunity may be beneficial in developing a more effective adaptive immune response against future variants.

Our study showed that RBD is primarily responsible for antibody neutralization while NTD likely acts in concert with RBD to enhance Ab neutralization. We showed that responses to RBD are most vulnerable to variant escape by Omicron BA.1. Our results are in line with research on monoclonal antibodies. Currently, almost all the therapeutic monoclonal Abs targets the RBD domain, particularly the receptor binding motifs. Most of them were subjected to Omicron escape (20). The ability of NTD Abs to act in concert with RBD for neutralization has also been shown in monoclonal Ab studies (67, 68). Unlike RBD monoclonal Abs that neutralizes infection by blocking ACE-2 interaction, NTD monoclonal Abs appeared to have little capacity to block ACE-2 interactions but can block S-mediated cell-cell fusion possibly through preventing interaction with auxiliary receptors (69).

We found that IgG1 and IgG2 correlated with Ab neutralization better than IgG3. The S1 (the major neutralizing domain) and S2 (the minor neutralizing domain) differentially utilize IgG1 and IgG3, respectively, which has been shown in our study as well as in COVID-19 patients (51). This difference may result in a poorer correlation of IgG3 with antibody neutralization as compared to that of IgG1. For RBD and S1, IgG1 and IgG2 but not IgG3 correlated significantly with ADCC. For S2, both IgG1 and IgG3 correlated strongly to ADCC. The data suggests an important role of S2 in eliciting IgG3 and ADCC antibodies.

In conclusion, our study showed that S1 and its subdomain RBD are antigenically superior for Ab neutralization and NK cell-mediated ADCC, however, they are subject to Omicron BA.1 escape, possibly due to higher frequencies of amino acid changes. The S2 domain showed comparable capacity to S1 in eliciting IgG3 and ADCC antibodies, and the responses were more resistant to Omicron BA.1 escape. Our results provide potential insights into mRNA vaccine immune mechanisms in protecting against severe disease in people who received standard two doses of mRNA vaccine and had little detectable neutralizing antibodies to Omicron BA.1. Additionally, our results suggest that S2 is a potential vaccine candidate against VOCs and perhaps other beta-coronaviruses.

The first limitation of the study is the inability to use the same samples in all the assays. Ideally, we would have used a matched sample set for all the experiments for each of the selected samples. However, we lacked the sample volume to perform these additional experiments since multiple assays had been previously performed using this cohort. To overcome the problem, the experiments were designed so that each figure should be able to stand alone to address one question. Sub-selection prioritized overlapping samples for those still have remaining volume but included non-overlapping samples to increase group size. Correlation analysis was performed on matched samples only.

Other limitations of this study are that the samples were obtained from subjects who received two doses of the mRNA vaccine, the lack of generalizability due to the homogeneity of the study group, and we did not compare the responses in people naturally infected, received booster doses or had breakthrough infections.

## Data availability statement

The raw data supporting the conclusions of this article will be made available by the authors, without undue reservation.

## Ethics statement

The studies involving humans were approved by Institutional Review Board, Naval Medical Research Center (NMRC IRB). The studies were conducted in accordance with the local legislation and institutional requirements. The participants provided their written informed consent to participate in this study.

## Author contributions

PS: Conceptualization, Data curation, Formal analysis, Supervision, Writing – original draft, Writing – review & editing, Project administration. CB: Conceptualization, Data curation, Formal analysis, Investigation, Writing – original draft, Writing – review & editing. LJ: Conceptualization, Formal analysis, Investigation, Methodology, Resources, Writing – original draft, Writing – review & editing. VJ: Data curation, Formal analysis, Investigation, Methodology, Writing – review & editing. YC: Data curation, Investigation, Writing – review & editing. ZZ: Investigation, Writing – review & editing. TB: Investigation, Writing – review & editing. QQ: Methodology, Writing – review & editing. TL: Data curation, Formal analysis, Writing – review & editing. MS: Funding acquisition, Supervision, Writing – review & editing. SJ: Funding acquisition, Resources, Supervision, Writing – review & editing. KC: Investigation, Resources, Writing – review & editing. NM: Investigation, Writing – review & editing. AL: Conceptualization, Investigation, Supervision, Writing – review & editing. RH: Conceptualization, Funding acquisition, Investigation, Project administration, Resources, Supervision, Writing – original draft, Writing – review & editing.
